# Beta-hydroxy-beta-methylbutyrate associated with low-intensity exercise training improves skeletal muscle regeneration through the IGF-Akt pathway

**DOI:** 10.1590/1414-431X2021e11597

**Published:** 2022-01-05

**Authors:** A.K. Yamada, R. Ferretti, C.Y. Matsumura, L. Antunes, C.A. da Silva, A. Pertille

**Affiliations:** 1 Programa de Pós-Graduação em Ciências do Movimento Humano, Laboratório de Plasticidade Neuromuscular, Universidade Metodista de Piracicaba, Piracicaba, SP, Brasil; 2 Departamento de Biologia Estrutural e Funcional, Instituto de Biociências de Botucatu, Universidade Estadual Paulista, Botucatu, SP, Brasil; 3 Instituto de Ciências da Saúde, Faculdade de Ciências da Saúde, Universidade Paulista - Swift, Campinas, SP, Brasil

**Keywords:** Supplementation, Regeneration, Muscle, Exercise, Injury, Signaling pathways

## Abstract

The effect of beta-hydroxy-beta-methylbutyrate (HMB) supplementation associated with exercise training at different intensities and frequencies on skeletal muscle regeneration of muscle-injured rats was investigated. Male Wistar rats were divided into sedentary and trained groups. The sedentary groups were subdivided into non-injured (SED-Ct), non-injured supplemented with HMB (SED-Ct-HMB), injured (SED), and injured with HMB (SED-HMB), and the trained groups were injured, supplemented with HMB, and then divided into training three times a week without load (HT3) or with load (HT3L) and training five times a week without load (HT5) and with load (HT5L). The rats received a daily dose of HMB associated with 60 min of swimming with or without 5% body mass load for 14 days. On the 15th day, cryoinjury was performed in the right tibialis anterior muscle (TA), and 48 h later, supplementation and training continued for 15 days. After the last session, the TA was dissected and a cross-sectional area (CSA) of muscle fibers was used to determine the percentage of CSA fibers and connective tissue (%CT), as well as the total and phosphorylated protein contents. SED-HMB showed increased CSA and decreased %CT and TGF-β when compared to SED. HT3 showed increased CSA and reduced %CT accompanied by increased IGF-1/Akt, myogenin, and MuRF1, and decreased TGF-β. The CSA of HT5L also increased, but at the cost of a higher %CT compared to the other groups. Our results demonstrated that HMB associated with training without load and with lower frequency per week may be a valuable strategy for skeletal muscle regeneration.

## Introduction

Skeletal muscle is a plastic tissue with exceptional regenerative capacity; only a few weeks after a severe injury that destroys fiber integrity, the structure and function of skeletal muscle can be completely restored (1). During the regeneration process, satellite cells play a role in cell proliferation, differentiation, and fusion to repair damaged fibers or compose new myofibers (1). Connective tissue remodeling is also an important step in muscle regeneration; a fibrogenic response acts as a scaffold for myofiber regeneration. However, post-injury excess connective tissue results in increased tissue scarring, which impairs muscle function (2).

Nutritional supplements have been widely investigated for their role in muscle regeneration (3,4), such as beta-hydroxy-beta-methylbutyrate (HMB), a metabolite of the amino acid leucine that increases the anabolic properties of protein synthesis in skeletal muscle (5) through the mammalian target of rapamycin (mTOR) pathway. This occurs via downstream p70^S6^ kinase (p70^S6K^) and decreases protein degradation through the ubiquitin-proteasome system (6). HMB has been shown to reduce exercise-induced muscle damage (7), activate proliferation of satellite cells during recovery from disuse atrophy (8), and minimize the inflammatory process (9). However, its effects on *in vivo* skeletal muscle regeneration in animal models such as rats or mice have not been studied. Supplementation with the HMB metabolite, leucine, after 10 days of cryolesion in muscle of young rats accelerates connective tissue repair and induces protein synthesis, but is not sufficient to promote an increase in the cross-sectional area (CSA) of regenerating myofibers (10). Thus, we can expect a potentiating effect of HMB in skeletal muscle regeneration, which is a potential new area of study in this field.

Exercise is a potential stimulus for muscle regeneration (11). Joanisse et al. (11) demonstrated that pre-conditioning treadmill running maximized skeletal muscle regeneration in aged rats. Richard-Bulteau et al. (12) also showed the recovery of muscle mass by subjecting rats to increased contractile activity during muscle regeneration, suggesting sustained action of myogenic cells. To our knowledge, no studies have yet associated HMB supplementation with exercise training in an *in vivo* muscle injury model. We hypothesized that HMB supplementation may increase muscle fiber regeneration in rats on different aerobic training protocols by regulating the insulin-like growth factor (IGF)-1-Akt pathway. Therefore, we aimed to investigate the effects of HMB supplementation in sedentary rats and determine the regulation processes of the IGF-1-Akt pathway signaling in muscle-injured rats supplemented with HMB and submitted to different intensities and frequencies of aerobic exercise training.

## Material and Methods

### Animals and experimental groups

**Figure 1:**
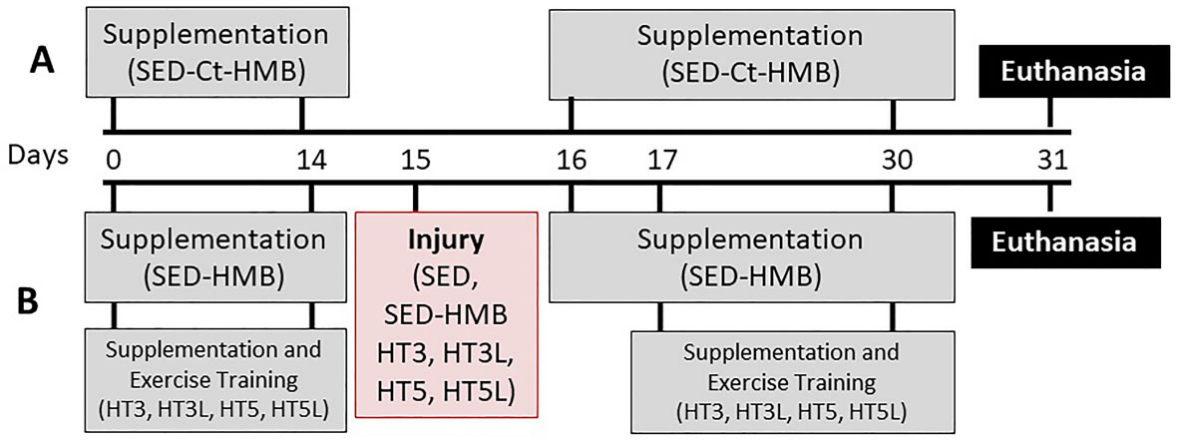
Schematic representation of the experimental protocol. All animals were subjected to cryoinjury in the right tibialis anterior muscle at the age of 24 weeks (day 15 of the protocol). **A**, Timeline of beta-hydroxy-beta-methylbutyrate (HMB) supplementation in sedentary non-injured (SED-Ct-HMB) rats. **B**, Timeline of the other study groups: SED: sedentary injured; SED-HMB: sedentary injured with HMB supplementation; HT3, HT3L: injured, supplemented, and 3 sessions per week of no-load or load training; HT5, HT5L: injured, supplemented, and 5 sessions per week of no-load or load training.

Thirty-six male Wistar rats (*Rattus novergucis*) were obtained and maintained at our institutional animal care facility at the College of Health Sciences of the Methodist University of Piracicaba (UNIMEP, Brazil). The rats were housed under controlled room temperature (23±2°C), 12-h light/dark cycles, and with free access to food and water. All procedures were performed following the guidelines of the Care and Use of Laboratory Animals, and the experimental protocols were approved by the UNIMEP Animal Care and Use Committee (#01/2015). All animals were subjected to cryoinjury in the right TA muscle at the age of 24 weeks (6 months) and were randomly distributed into two experimental designs, one with sedentary animals and the other with trained animals. Sedentary animals (n=12) did not undergo any type of training and were divided into the following groups: sedentary with normal diet (n=6) and sedentary supplemented with HMB (n=6). For sedentary animals, the left (contralateral, without injury) TA muscles were used as a control for the right (cryoinjured) TA muscles, forming four groups: i) non-injured sedentary control (Sed-Ct); ii) non-injured sedentary control supplemented with HMB (Sed-Ct-HMB); iii) injured sedentary with normal diet (Sed); and iv) injured sedentary supplemented with HMB (Sed-HMB; Figure 1A and B).

Trained animals (n=24) were supplemented with HMB and divided into four experimental groups according to the frequency and intensity of training: i) 3 sessions per week of no-load training (HT3; n=6); ii) 3 sessions per week of load training (HT3L; n=6); iii) 5 sessions per week of no-load training (HT5; n=6); and iv) 5 sessions per week of load training (HT5L; n=6; Figure 1B).

### HMB supplementation

The animals from the Sed-Ct-HMB, Sed-HMB, HT3, HT3L, HT5, and HT5L groups were supplemented daily with HMB (320 mg·kg^-1^·day^-1^; Arnold Nutrition Inc., USA). The dose was based on Pimentel et al. (13) and HMB supplementation was given by gavage 1 h before the training protocol (Figure 1A and B).

### Exercise training

An adapted rectangular aquarium tank (100×40×50 cm) with controlled water temperature (30±2°C) was used for exercise training. Animals were previously adapted to the water environment. On the first day, the animals were placed in shallow water for 30 min for familiarization. On the second day, they performed 10 min of swimming; on the third day, they performed 20 min of swimming; on the fourth day, they performed 20 min of swimming with or without load according to the intervention group; and on the fifth day, they performed 30 min of swimming with or without load according to the intervention group. Five percent of the animal’s body weight was used as load and considered as an indicator of effort intensity. According to Gobatto et al. (14), lactate stabilizes at this intensity, providing a balance between its accumulation and removal rates. The load was adjusted weekly according to alterations in body weight measured by a calibrated digital scale (Gehaka, BG 1000, Brazil).

Each training session was performed for 1 h and over 14 days as part of the muscle preconditioning phase before the cryoinjury protocol was implemented. The cryoinjury protocol was performed on day 15. After a 48-h recovery period, exercise training was continued for 14 days, providing a total of 28 days of training and 30 days for the entire protocol (Figure 1B).

### Muscle injury procedure

On day 15 of the protocol, animals were anesthetized via intraperitoneal injection with a mixture of ketamine hydrochloride and xylazine hydrochloride (1:1 ratio) and 0.3 mL/100 g dose of body weight. After signs of anesthesia appeared, the injury area was trichotomized and the right TA muscle was exposed. The cryoinjury was induced by pressing an iron bar (1×0.5 cm) cooled in liquid nitrogen for 30 s against the muscle belly for 10 s twice, according to Miyabara et al. (15) protocol. Following these procedures, the muscle fascia and skin were sutured, and the animals were allocated individually in cages with food and water *ad libitum* for recovery (48 h).

### Animal euthanasia

At the end of the experimental period (on day 31), the animals were anesthetized as described above, and after clear signs of general anesthesia, the right TA muscle of all experimental groups and the left TA of sedentary experimental groups were removed, weighed, and transversely divided in the center of the muscle into two proportional cross-sections, one used for light microscopy and the other used for immunoblotting. For histological analysis, the entire half of the muscle was positioned to obtain the cross-sections. For the western blot (WB) analysis, the other half of the right TA muscle was reduced, including the injured region.

### Morphometrical parameters

Muscles were frozen and cross-sectioned into 8-µm thick slices using a cryostat (model HYRAX C 25, Zeiss, Germany) and stained with hematoxylin and eosin. The slides were assembled with Entellan (Merck, Germany) and the cuts were used to measure the CSA and the density of connective tissue (CT). For the CSA measurement, approximately 12 random fields per slide were used, one slide for each animal. Two hundred and fifty fibers with centralized nuclei in the right TA (regenerated fibers) were analyzed in all injured muscles, and 250 fibers with peripheral nuclei (normal fibers) were analyzed in the left TA (non-injured muscle) of sedentary groups using a light microscope (Nikon Eclipse E 400, Japan) with a 20× objective-coupled video camera (Nikon Express Series) connected to a computer with the Image Pro-Plus^®^ 6.0 software (Media Cybernetics, USA).

CT measurement was performed using Image-Pro Express software with images acquired through a video camera (Nikon Express Series) coupled to a light microscope with 20× objective (Nikon Eclipse E 400). Six images for the right TA were analyzed and featured a grid containing 140 intersecting lines, which were counted and superimposed on the connective tissue; the results were transformed into percentages (16).

### Western blot analysis

The muscles were homogenized in buffer (1% Triton X-100, 100 mM Tris-HCL, pH 7.4, 100 mM sodium pyrophosphate, 100 mM sodium fluoride, EDTA 10 mM sodium orthovanadate, 2 mM PMSF, and 0.1 mg mg/mL aprotinin). The extracts were centrifuged at 13,552 *g* at 4°C for 20 min, and the soluble fraction was treated with Laemmli buffer (0.1% bromophenol blue and 1 M sodium phosphate pH 7.0, 50% glycerol, and 10% sodium dodecyl sulfate [SDS]) plus 100 mM dithiothreitol and placed in a heated dry bath for 5 min. Then, 60 µg of protein was applied on SDS-polyacrylamide gel with 12% of the protein placed in the apparatus for electrophoresis (Bio-Rad Laboratories, USA).

Nitrocellulose membrane gel electrotransfer was performed within 90 min at 120 V. The membranes were washed with a basal solution (10 mM base Tris, 150 mM sodium chloride and Tween) and incubated with 10 µg of primary antibody to myogenin (monoclonal mouse; Sigma Aldrich, M5815, USA), TGF-β1 (mouse monoclonal antibody; Sigma-Aldrich, T7039), IGF-1 (polyclonal rabbit antibody; Bioss Antibodies, bs-4588R, USA); phospho-Akt (Ser473, monoclonal rabbit antibody; Cell Signaling Technology, 4060, USA); phospho-mTOR-Ser2448 (monoclonal rabbit antibody; Millipore, 04-385, USA), total-mTOR (polyclonal rabbit antibody; Cell Signaling Technology, 2972, USA), p70^S6K^ kinase (monoclonal rabbit antibody; Cell Signaling Technology, 2708); myostatin (rabbit polyclonal antibody; GDF8/MSTN; Bioss Antibodies, bs-1288R); FoxO3a (monoclonal rabbit antibody; Cell Signaling Technology, 12829, USA); MAFbx (monoclonal mouse antibody; Santa Cruz Biotechnology, sc-166806, USA); MuRF1/Trim63 (polyclonal rabbit antibody; Bioss Antibodies, bs-2539R, USA), and GAPDH (polyclonal rabbit antibody; Santa Cruz, sc-25778, USA) diluted in 10 mL of a basal solution containing 3% skim milk at 4°C. The following day, they were washed for 30 min with a basal solution containing 3% skim milk and 2.5 µg of secondary antibody (goat anti-rabbit IgG-horseradish peroxidase [HRP]; Santa Cruz, sc-2004 or goat anti-mouse IgG-HRP, Santa Cruz, sc-2005, USA) for 2 h at room temperature, then washed again for 30 min with basal solution.

To determine the immunoreactive bands, the membranes were exposed to the chemiluminescent solution Super Signal West Pico Chemiluminescent, Pierce, USA, for 5 min, and then the fluorescent signal was captured on the G-box (GeneSys, Syngene, USA ) equipment. Data are reported as arbitrary units, obtained by dividing the level of the protein under study by the level of GAPDH (internal control protein).

### Statistical analysis

Data analysis was performed using GraphPad Prism software version 5.0 (USA). Normality was verified using the Shapiro-Wilk test, and comparisons between the training groups were performed using one-way analysis of variance (ANOVA) and Tukey’s test. For all tests, a significance value of P≤0.05 was considered.

## Results

### Effects of HMB in sedentary rats

**Figure 2:**
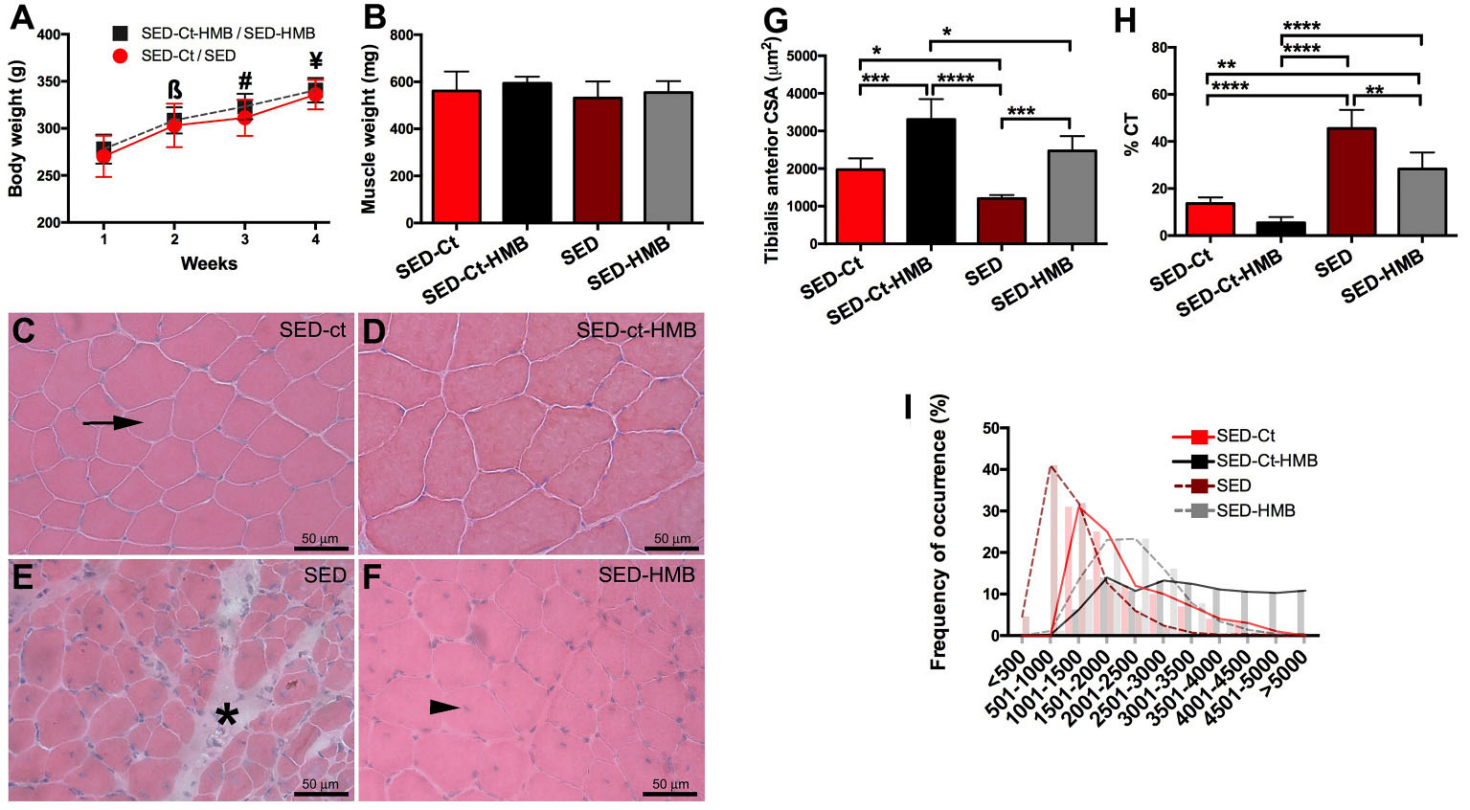
Beta-hydroxy-beta-methylbutyrate (HMB) in sedentary rats. Comparison between sedentary non-injured (SED-Ct), sedentary non-injured supplemented with HMB (SED-Ct-HMB), injured sedentary (SED), injured sedentary with HMB (SED-HMB) on body weight (**A**), muscle weight (**B**), histological cross-sections of the tibialis anterior muscle stained with HE (**C**-**F**, scale bar 50 µm), cross-sectional area (CSA) (**G**), connective tissue (CT) percentage (**H**), and frequency of muscle fibers according to cross-sectional area in µm^2^ (**I**). Continuous lines represent SED-Ct and SED-Ct-HMB and dashed lines represent SED and SED-HMB. The arrow in **C** indicates a fiber with peripheral nuclei, the arrowhead in **F** indicates a fiber with centralized nuclei, and the asterisk (*) in **E** indicates connective tissue. Data are reported as means±SD. ^?^P<0.05 *vs* 1st week SED Ct/SED, ^#^P<0.05 *vs* 2nd week SED-Ct/SED, ^¥^P<0.05 *vs* 3rd week SED-Ct/SED (Student’s *t*-test). *P<0.05, **P<0.01, ***P<0.001, ****P<0.0001 (one-way ANOVA and Tukey’s test).

After 4 weeks, the body weight increased in all sedentary groups (Figure 2A). The muscle weight did not increase in the HMB supplemented groups (Figure 2B). The muscle fibers of the SED-Ct and SED-Ct-HMB groups had a polygonal shape with peripheral nuclei and a small amount of extracellular matrix, which are characteristics of normality (Figure 2C and D). In the injured groups (SED and SED-HMB), muscle fibers had centralized nuclei, which characterizes the regeneration process (Figure 2E and F). In the SED group, the presence of connective tissue can be noted (Figure 2E).

HMB increased the CSA in SED-Ct-HMB compared to the other groups. In contrast, SED showed a lower CSA and a higher %CT compared with the other groups (Figure 2G and H, respectively). Additionally, HMB increased the distribution in muscle fiber diameter; SED-HMB had a higher number of regenerated muscle fibers with diameters of 2,000 and 4,000 µm and SED-Ct-HMB had the highest muscle fiber diameters, ranging from 2,000-5,000 µm (Figure 2I).

**Figure 3:**
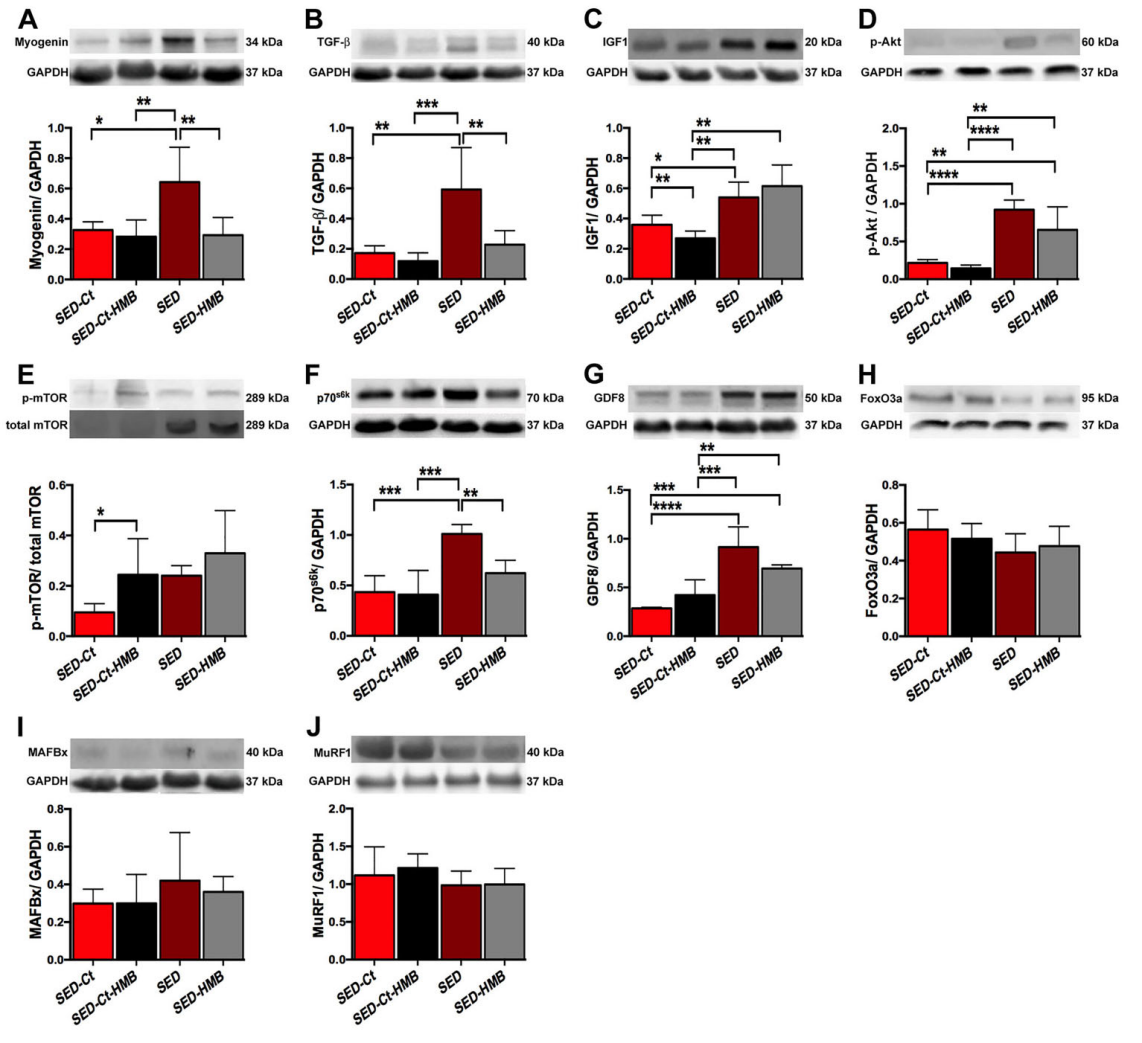
Western blot analysis of sedentary non-injured (SED-Ct), sedentary non-injured supplemented with HMB (SED-Ct-HMB), sedentary injured (SED), sedentary injured with HMB (SED-HMB) rats. Protein levels of myogenin (**A**), TGF-β (**B**), IGF-1 (**C**), phospho-Akt (**D**), p-mTOR/t-mTOR (**E**), p70^S6K^ (**F**), GDF8 (**G**), FoxO3a (**H**), MAFbx (**I**), and MuRF1 (**J**); n=6 rats in each group. Data are reported as means±SD. *P<0.05, **P<0.01, ***P<0.001, ****P<0.0001 (one-way ANOVA and Tukey’s test).

With respect to signaling pathways, the SED group presented a significant increase in myogenin and TGF-β levels compared to the other groups (Figure 3A and B, respectively). The SED and SED-HMB groups showed a significant increase in IGF-1(Figure 3C) and p-Akt (Figure 3D) compared to the Ct groups. The ratio between phosphorylated mTOR (p-mTOR) and total mTOR showed a significant increase only in the SED-Ct-HMB group compared to the SED-Ct group (Figure 3E). The p70^s6k^ level was elevated only in the SED group (Figure 3F). The injured muscles in the sedentary animals (SED and SED-HMB) showed a significant increase in GDF8 (Figure 3G) compared to non-injured muscles (SED-Ct and SED-Ct-HMB). There were no differences in FoxO3a (Figure 3H), MAFbx (Figure 3I), and MuRF1 (Figure 3J) levels between sedentary groups.

### Effects of HMB in different exercise protocols

**Figure 4:**
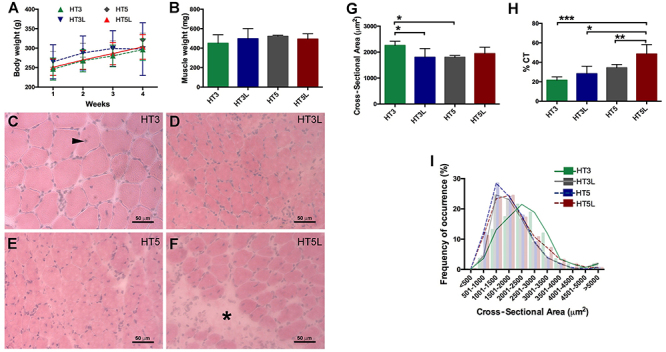
Effect of beta-hydroxy-beta-methylbutyrate (HMB) on injured rats submitted to different exercise protocols on body weight (**A**), muscle weight (**B**), histological cross-sections of tibialis anterior muscle stained with HE (**C**-**F**, scale bar 50 μm), cross-sectional area (**G**), connective tissue (CT) % (**H**), and muscle fiber distribution according to cross-sectional area in µm^2^ (**I**). HT3, HT3L: injured, supplemented, and 3 sessions per week of no-load or load training; HT5, HT5L: injured, supplemented, and 5 sessions per week of no-load or load training. The arrowhead in **C** indicates a fiber with centralized nuclei and the asterisk (*) in **F** indicates connective tissue. Data are reported as means±SD. *P<0.05, **P<0.01, ***P<0.001 (one-way ANOVA and Tukey’s test).

Over the course of 4 weeks, the animals’ body weight increased in all four protocols: HT3, HT3L, HT5, and HT5L (Figure 4A), even though the muscle weight did not change among the groups (Figure 4B). All trained groups had muscle fibers with centralized nuclei, which characterizes the regeneration process (Figure 4C-F). There was also an increase in connective tissue in the HT5L group (Figure 4F). We observed a significant decrease in CSA in the HT3L and HT5 groups compared with the HT3 group (Figure 4G) and a significant increase in connective tissue in the HT5L group compared with the other three groups (Figure 4H). Furthermore, we observed an anabolic effect of HMB in HT3 by measuring the distribution in muscle fiber cross-sectional area (Figure 4I).

**Figure 5:**
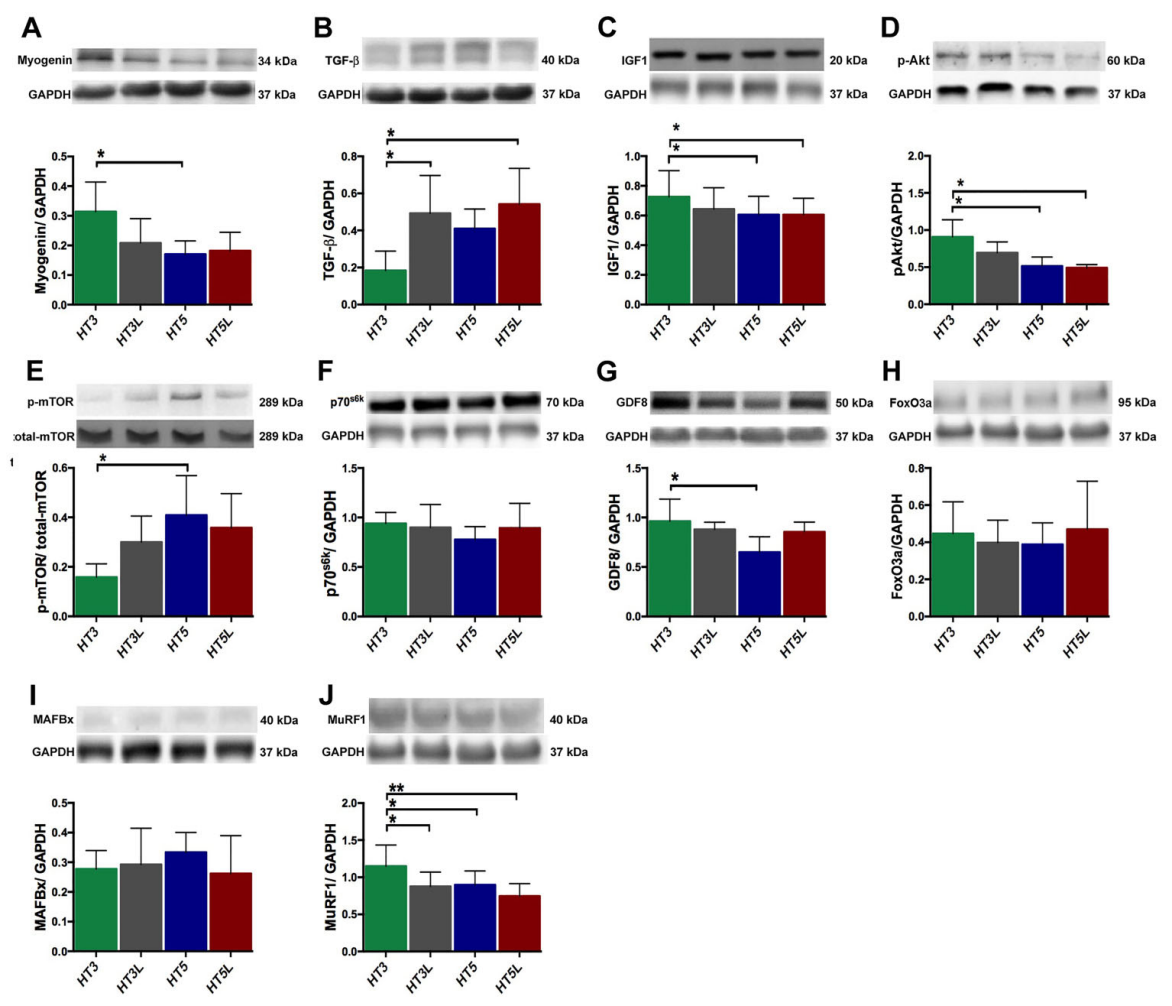
Western blot analysis in beta-hydroxy-beta-methylbutyrate (HMB) supplemented rats in different exercise protocols. Protein levels of myogenin (**A**), TGF-β (**B**), IGF-1 (**C**), p-Akt (**D**), p-mTOR/t-mTOR (**E**), p70^S6K^ (**F**), GDF8 (**G**), FoxO3a (**H**), MAFbx (**I**), and MuRF1 (**J**); n=6 rats in each group. HT3, HT3L: injured, supplemented, and 3 sessions per week of no-load or load training; HT5, HT5L: injured, supplemented, and 5 sessions per week of no-load or load training. Data are reported as means±SD. *P<0.05, **P<0.01 (one-way ANOVA and Tukey’s test).

In the signaling pathways, HT3 had increased myogenin compared with the HT5 group and decreased TGF-β levels compared with the HT3L and HT5L groups (Figure 5A and B, respectively). HT3 had significantly increased IGF-1 and p-Akt compared to HT5 and HT5L (Figure 5C and D) and a reduced p-mTOR level compared to HT5 group (Figure 5E). There were no statistical differences in p70^S6K^ among groups (Figure 5F). Finally, we observed no differences in FoxO3a and MAFbx expression among training groups (Figure 5H and I). Although the normalized GAPDH protein level of MuRF was significantly decreased in all groups compared to the HT3 group (Figure 5J), the protein level of GDF8 was significantly increased in the HT3 group compared to the HT5 group (Figure 5G).

## Discussion

To our knowledge, this was the first study to investigate the combination of HMB and different intensities of aerobic exercise training in the regeneration of skeletal muscle injuries in rats. We evaluated the effects of the interventions 16 days after muscle injury because at this stage the muscle is still in the regeneration process. Although a complete TA regeneration has been reported after 21 days of injury (17), we observed that HMB supplementation significantly increased the muscle CSA and decreased the connective tissue area.

HMB seems to act differently on injured and non-injured muscles after 29 days of supplementation. The SED-Ct-HMB (non-injured) muscles showed an increased TA CSA and a significant increase in the level of p-mTOR, but there were no differences in the level p70^S6K^ compared to the SED-Ct, as observed in a previous study that evaluated sedentary and healthy rats supplemented with HMB for a month. In that study, the intervention directly induced a significant increase in mTOR expression and in the phosphorylation of p70^S6K^, but the p70^S6K^ expression was not altered (13).

The SED-HMB group (with injury) showed an increase in the TA CSA compared with the SED groups and, in both injured groups there was a significant increase in IGF-1 and p-Akt levels compared to the non-injured groups. IGF-1/Akt not only contributes to muscle regeneration (18,19), but is also associated with skeletal muscle hypertrophy (20,21), thus corroborating our findings of IGF-1/Akt pathway activation.

HMB supplementation (SED-HMB) decreased fibrosis during the regeneration process, mainly due to significantly decreased TGF-β1 levels. With respect to fibrosis of skeletal muscle, TGF-β1, a fibrogenic factor, regulates the phenotype and function of fibroblasts as a key role in myofibroblast transdifferentiation (22). TGF-β1 inhibition leads to muscle regeneration by reducing fibrosis (23), suggesting that HMB may have blunted the development of fibrosis. Therefore, HMB supplementation accelerates connective tissue repair of injured TA muscle (24).

Exercise minimizes the development of connective tissue in regenerating muscles (25). Our results showed that HMB supplementation for 3 days and training without load (HT3) increased myogenin and decreased TGF-β1. Although the CSA of HT3 and HT5L were not statistically different, the HT5L showed increased %CT, which indicates fibrosis. One possible explanation is that low-intensity exercise may have prevented fibrosis (26). Rats that have been exercised for 14 days before injury and 14 days after injury showed a reduced deposition and expression of collagen I (25). However, to our knowledge, only one study has compared the effects of different exercise intensities on the extracellular matrix of skeletal muscle. One study demonstrated that high-intensity exercise in rats induced an increase in matrix metalloproteinase 2 (MMP-2), demonstrating that treadmill running at fast speeds leads to expression of MMP-2, indicating accelerated activity of the active form of MMP-2 and increased extracellular matrix degradation (27).

In parallel with the increased CSA in the HT3 group (which is associated with a higher number of muscle fibers with a diameter between 2,000 and 4,000 µm in diameter) compared to the HT3L and HT5 groups, HT3 group had increased protein levels of IGF-1 and Akt compared to the HT5 and HT5L groups. The difference between the HT3 and HT5 groups may be due to increased regeneration capacity of the HT3 group, assessed by the increase in the CSA of muscle fibers with centralized nuclei (28). Evidence suggests that muscle hypertrophy is a cellular indicator of muscle regeneration (11). Also, the HT3 group presented with increased levels of myogenin compared to the HT5 group. Myogenin is an important marker of regeneration, regulating myocyte fusion and determining the number of muscle fibers (29). HMB also increases IGF-1 mRNA levels and accelerates cell function (30); therefore, we suggest that HMB associated with no-load low-frequency training triggers improvements in muscle adaptation in response to cryoinjury.

Treadmill and voluntary exercises were shown to lead to the recovery of muscle mass and CSA of notexin-injured muscles after 21 days (11,12), with an increase in MyoD and myogenin and mTOR pathway activation during the initial stages of regeneration (12). Another study found that non-strenuous exercise (voluntary wheel running) enhanced old muscle repair, in part by repressing TGF-β levels (31). While these studies found that exercise improves regeneration, they did not compare different exercise intensities, underscoring the novelty of our study design.

The mTOR response has been shown to be sensitive to training volume (32,33). Although we did not observe increased CSA in HT5, mTOR protein levels were increased compared with HT3, suggesting that training volume (in our case, frequency) may have stimulated this effect. Further studies exploring the association between endurance training and the mTOR pathway will elucidate the answers to these intriguing questions.

We observed increased GDF8 (myostatin) protein levels in the HT3 group compared to the HT5 group, as well as increases in the injured groups (SED and SED-HMB) compared to the non-injured groups. The study of the myostatin/activin type I receptor (ALK4) showed that one of the key receptors of the myostatin pathway influences myogenesis and regulates the tight balance between protein synthesis and degradation to maintain muscle mass during the regeneration process (34).

We also analyzed muscle RING finger 1 (MuRF1) and muscle atrophy F-box (MAFbx) as markers of muscle atrophy (35). The expression of MuRF1 and MAFbx rapidly increases in response to a variety of stressors including unloading, decreased neural activity, elevated glucocorticoids, elevated cytokines, increased oxidative stress, and malnutrition (35). In skeletal muscle, FoxO3a induces muscle atrophy in response to disuse and metabolic stress (36). We are aware that other muscle signaling pathways should be considered. AMPK (AMP-activated protein kinase) is involved in muscle contraction and muscle adaptation from exercise (37), as endurance training results in oxidative phenotype in muscle tissue, increasing the expression of proteins involved in metabolism and mitochondria enzymes (38). In addition, the absence of AMPK in satellite cells disrupts muscle regeneration after injury, indicating that adequate levels of AMPK must be maintained for optimal muscle regeneration (39). Among other functions, AMPK promotes protein degradation, which, when phosphorylated, increases the expression of MAFbx and MuRF1 (40); this could explain the increase in MuRF1 in the HT3 group.

Limitations of the study include the lack of baseline assessments showing the homogeneity between experimental groups before the intervention and measures of physical capacity in the training groups. The lack of a single trained group (without supplementation) is also a limitation. Even if no variation in GADPH was found, the use of another normalizer can be of interest.

We used an injury model that requires protein analysis at different time points. A detailed molecular screening of skeletal muscle regeneration in the long term, as well as the evaluation of functional muscle parameters, will provide a deeper understanding of skeletal muscle regeneration and the characterization of other putative signaling pathways. Other models of muscle injury, different training regimens, different doses of HMB, and the use of multi-OMICS approaches will also be of interest.

In conclusion, this study demonstrated that HMB supplementation improved muscle regeneration. The association with high-intensity and high-frequency exercise protocols significantly increased skeletal muscle hypertrophy at the expense of increased fibrosis. Moreover, low-intensity (without load) and low-frequency (three times a week) exercise training associated with HMB appeared to be the most effective protocol for improving skeletal muscle regeneration in this muscle injury rat model.
